# Genome Sequence of a Gamma- and UV-Ray-Resistant Strain, *Deinococcus wulumuqiensis* R12 

**DOI:** 10.1128/genomeA.00206-13

**Published:** 2013-05-09

**Authors:** Xian Xu, Ling Jiang, Zhidong Zhang, Yuhu Shi, He Huang

**Affiliations:** State Key Laboratory of Materials-Oriented Chemical Engineering, College of Biotechnology and Pharmaceutical Engineering, Nanjing University of Technology, Nanjing, People’s Republic of Chinaa;; Institute of Microbiology, Xinjiang Academy of Agricultural Sciences, Urumqi, Xinjiang Uigur Autonomous Region, People’s Republic of Chinab

## Abstract

*Deinococcus wulumuqiensis* R12, isolated from radiation-polluted soil, is a red-pigmented strain of the extremely radioresistant genus *Deinococcus*. It contains a major carotenoid, namely, deinoxanthin. Here, we present a 3.39-Mb assembly of its genome sequence, which might provide various kinds of useful information related to *Deinococcus*, such as about the key enzymes of its radioresistance mechanism and carotenoid biosynthetic pathways.

## GENOME ANNOUNCEMENT

The genus *Deinococcus* is particularly interesting since a large number of its members are extremely radioresistant (1, 2). *Deinococcus radiodurans* R1 was the first of the deinobacteria to be discovered and was isolated from canned meat that had spoiled following exposure to X rays (3). This strain shows extraordinary resistance to ultraviolet and gamma radiation, desiccation, hydrogen peroxide, and many other DNA-damaging agents (4, 5). For example, the resistance to UV irradiation of *D. radiodurans* is 20 times higher than that of *Escherichia coli*, while the resistance to ionizing radiation is 200 times higher than that of *E. coli* (6).

We recently characterized a new bacterial species, *Deinococcus wulumuqiensis* strain R12, which showed that it warrants recognition as a novel species in the genus *Deinococcus*. It was isolated from the radiation-contaminated soils in the Xinjiang Uigur Autonomous Region of northwest China. Strain R12 is a Gram-positive, reddish orange, non-spore-forming coccus, with gamma radiation resistance to >10 kGy and UV resistance to >700 J m^-2^ (7). This strain shows a higher tolerance for gamma radiation and UV light than does *D. radiodurans* R1. R12 is red pigmented because of its carotenoid biosynthetic abilities for cellular protection (8). However, its carotenoid biosynthetic pathways and radioresistance mechanism are not clear. The resistance of R12 and its biosynthetic pathways might be the result of multiple closely coordinated mechanisms involving numerous proteins. Studies that aim to understand these detailed mechanisms are now focused on the analysis and annotation of a complete genome sequence.

Here, we present the draft genome sequence of strain R12, obtained using the Illumina HiSeq 2000 next-generation DNA platform. Sequencing was performed by Shanghai Majorbio Pharm Technology Co., Ltd., with a paired-end library. The reads were trimmed and *de novo* assembled with SOAP*denovo* (http://soap.genomics.org.cn/soapdenovo.html). Open reading frames (ORFs) were identified by the program Glimmer (http://www.cbcb.umd.edu/software/glimmer/). These ORFs were further annotated by comparison with the NCBI NR database and BLASTp (BLAST 2.2.24). The rRNAs were predicted by RNAmmer (9), and tRNAs were predicted by tRNAscan (10).

The draft genome sequence of strain R12 comprises 3,391,664 bp, which is assembled into 239 contigs. The *N*_50_ quality measurement of the contigs is 31,304 bp, with an average contig size of 23 kb, and the largest contig assembled is approximately 133 kb. It has a G+C content of 66.38%. There are 3,160 predicted protein-coding sequences in the genome sequence. The chromosome has 3 rRNA operons and 45 tRNAs as predicted by RNAmmer and tRNAscan, respectively.

The genome sequence of strain R12 serves as a foundation for further investigation of the molecular basis of its resistance to DNA-damaging agents. Further analysis of the genome sequence might also provide other useful information related to R12, such as identifying the genes that are involved in its radioresistance mechanism and carotenoid biosynthetic pathways.

### Nucleotide sequence accession numbers.

This Whole-Genome Shotgun project has been deposited at DDBJ/EMBL/GenBank under the accession no. APCS00000000. The version described in this paper is the first version, accession no. APCS01000000.

## References

[1] Asker D Awad TS Beppu T Ueda K 2008 *Deinococcus misasensis* and *Deinococcus roseus*, novel members of the genus *Deinococcus*, isolated from a radioactive site in Japan. Syst. Appl. Microbiol. 31:43–49 18096345 10.1016/j.syapm.2007.10.002

[2] Brooks BW Murray RGE 1981 Nomenclature for “*Micrococcus radiodurans*” and other radiation-resistant cocci: *Deinococcaceae* fam. nov. and *Deinococcus* gen. nov., including five species. Int. J. Syst. Bacteriol. 31:353–360

[3] Anderson AW Nordan HC Cain RF Parrish G Duggan D 1956 Studies on a radoresistant micrococcus. I. Isolation, morphology, cultural characteristics, and resistance to gamma radiation. Food Technol. 10:575–578

[4] Agostini HJ Carroll JD Minton KW 1996 Identification and characterization of uvrA, a DNA repair gene of *Deinococcus radiodurans*. J. Bacteriol. 178:6759–6765 8955293 10.1128/jb.178.23.6759-6765.1996 PMC178572

[5] Meima R Lidstrom ME 2000 Characterization of the minimal replicon of a cryptic *Deinococcus radiodurans* SARK plasmid and development of versatile *Escherichia coli*–*D*. *radiodurans* shuttle vectors. Appl. Environ. Microbiol. 66:3856–3867 10966401 10.1128/aem.66.9.3856-3867.2000 PMC92231

[6] White O Eisen JA Heidelberg JF Hickey EK Peterson JD Dodson RJ Haft DH Gwinn ML Nelson WC Richardson DL Moffat KS Qin H Jiang L Pamphile W Crosby M Shen M Vamathevan JJ Lam P McDonald L Utterback T Zalewski C Makarova KS Aravind L Daly MJ Minton KW Fleischmann RD Ketchum KA Nelson KE Salzberg S Smith HO Venter JC Fraser CM 1999 Genome sequence of the radioresistant bacterium *Deinococcus radiodurans* R1. Science 286:1571–1577 10567266 10.1126/science.286.5444.1571 PMC4147723

[7] Wang W Mao J Zhang Z Tang Q Xie Y Zhu J Zhang L Liu Z Shi Y Goodfellow M 2010 *Deinococcus wulumuqiensis* sp. nov., and *Deniococcus xibeiensis* sp. nov., isolated from radiation-polluted soil. Int. J. Syst. Evol. Microbiol. 60:2006–2010 19801390 10.1099/ijs.0.015917-0

[8] Tian B Hua Y 2010 Carotenoid biosynthesis in extremophilic *Deinococcus*–*Thermus* bacteria. Trends Microbiol. 18:512–520 20832321 10.1016/j.tim.2010.07.007

[9] Lagesen K Hallin P Rødland EA Stærfeldt HH Rognes T Ussery DW 2007 RNAmmer: consistent and rapid annotation of ribosomal RNA genes. Nucleic Acids Res. 35:3100–3108 17452365 10.1093/nar/gkm160 PMC1888812

[10] Lowe TM Eddy SR 1997 tRNAscan-SE: a program for improved detection of transfer RNA genes in genomic sequence. Nucleic Acids Res. 25:955–964 9023104 10.1093/nar/25.5.955 PMC146525

